# People and bats in Fiji: perceptions and interactions

**DOI:** 10.1098/rsos.250383

**Published:** 2026-11-04

**Authors:** Jessica L. McCutchan, Alivereti Naikatini, David T. S. Hayman, Brett D. Gartrell

**Affiliations:** ^1^ Massey University, Palmerston North, New Zealand; ^2^ The University of the South Pacific, Suva, Central Division, Fiji

**Keywords:** Oceania, Pacific, mixed methods, conservation, infectious disease, zoonotic disease, human–wildlife interaction

## Abstract

The management of wild bat populations often presents challenges in balancing the needs of biodiversity conservation and public health risks from zoonotic diseases. In Fiji, the most important recognized zoonotic infection is *Leptospira*, which was recently detected in wild bat populations. Five out of six species of Fijian bats are considered threatened to critically endangered on the IUCN Red List. This study investigates the perceptions of bats by people in Fiji and aims to identify and quantify the reported frequency and types of interactions between bats and people. In this mixed-methods study, 198 Fijian people were interviewed across the three main islands of Fiji: Viti Levu, Vanua Levu and Taveuni. Bats were generally perceived positively (63%, 125/198), mostly because they are perceived to be ‘clean, healthy and tasty’ meat sources (73%, 134/184). The majority of participants consume and come into contact with bats or with their urine, faeces, blood and saliva. Older adults, men and those who recognize that bats can cause harm are more likely to contact bats in Fiji. Quantifying these interactions and identifying other risk factors for bat exposure is an important step in targeting conservation efforts, community engagement and education, as well as determining at-risk groups for possible zoonotic pathogen exposure.

## 1. Introduction

People's perceptions and beliefs influence their interactions and behaviours, and this is reflected in wildlife–human interactions, such as through hunting, consumption, avoidance or care, which, in turn, shape conservation and public health measures [1–3]. Studying and understanding these perceptions and interactions can lead to improved wildlife conservation efforts, improved wildlife and human health, reduction of human–wildlife conflicts and a deeper understanding of community health and growth [4–7].

Wildlife is an important and often overlooked reservoir for zoonotic pathogens, and an increase in human–wildlife contact results in a higher risk of infection if a pathogen is present [8,9]. Bats (order Chiroptera) are known reservoirs for some pathogens or their ancestors [10–23]. In many cases, transmission is known or suspected to occur via an intermediate host with which humans come into contact and become infected; however, direct transmission of zoonotic pathogens from bats to humans is also possible (e.g. rabies virus in South America) [24–26]. The Pacific Island countries and territories are reported to be free of the most serious wildlife-associated zoonotic diseases which are prevalent in other parts of the globe, such as highly pathogenic avian influenza and rabies [27]. However, there is limited scientific evidence to confirm this animal health status, despite the recognized vulnerability of the Pacific Islands to climate change effects on ecosystems and public health [28].

In Fiji, most attention and research have focused on domestic animals as reservoirs for the most common zoonoses, such as leptospirosis [29], bovine tuberculosis [30] and arboviruses (Ross River virus) [31]. However, we recently discovered evidence of *Leptospira* in Fijian bats [32], which suggests wildlife should be considered in the epidemiology of this zoonosis. Recent metagenomic analyses of isolated bat populations of <2600 individuals suggest that viral diversity might be limited on some isolated oceanic islands [33]. However, metagenomic analysis of faeces from Pacific flying foxes (*Pteropus tonganus*) in Tonga, a species also present in Fiji, suggests these animals have a diverse virome [34,35]. Moreover, an outbreak of henipavirus in the Philippines [36] and serological evidence of henipavirus in Filipino [37] and Papua New Guinean bats [38] would suggest that potentially zoonotic viruses may be present among these Old World bats, as seen on other island systems [39–43].

Bats are key bioindicator taxa given their ecological importance and vulnerability to natural and anthropogenic changes [44]. The conservation of all bat species is important in itself for the maintenance of stable ecosystems and for their economic value, whether directly through human consumption or indirectly through seed dispersal and pollination of many plants, and pest control within agricultural crops [45–49]. Globally, over a third of bat species assessed by the International Union for Conservation of Nature (IUCN) are considered threatened or data deficient, and well over half of bat species have unknown or decreasing population trends [31]. In Fiji, 96% of the plant species serviced by bats are valued by humans for economic, medicinal and cultural uses [50]. As five of the six species of bats in Fiji are listed from Near Threatened through to Critically Endangered on the IUCN Red List, it is evident that conservation of all species is critical to preserving both Fijian biodiversity and the ecosystem services on which local communities depend [51–56]. Population monitoring studies have identified a decrease in roosting sites and other possible threats to the Fijian bat population. Few studies, however, have explored the details of how human–bat interactions, and the perceptions surrounding bats may impact conservation efforts [57–61].

Community engagement with wildlife has recently become a key focus point for many studies of wildlife conservation [3,62–64]. By actively involving local communities in the management, monitoring and stewardship of nearby wildlife, conservation efforts can be more successful and more likely to be sustained over time [62,63,65]. Gaining a more comprehensive understanding of the community demographics, current perceptions and values of wildlife provides better insight towards informed project design and realistic conservation goals [62,66,67]. People can have negative perceptions of bats, which subsequently have a negative impact on conservation efforts of the order, such as in Kenya [68], where some respondents reported bats as ‘agents of the devil, which could be sent by witch doctors to harm local people’. Rego and colleagues [69] studied the interactions and ideas surrounding bats in villages on the border of a biological reserve in northern Brazil. Over 90% of participants perceived bats negatively. There was a strong belief that bats caused harm and disease, although some participants mentioned their use of bats in traditional medicine. In the Asia-Pacific, however, bats are perceived positively by the majority of cultures [70].

In the Cook Islands, Cousins & Compton researched human–bat interactions, hunting pressures on *P. tonganus* for consumption, as well as the perceptions from local communities (both adults and children) [71]. The study highlighted areas for targeted education efforts and identified groups interested in learning more and being involved in conservation efforts, as well as the need for (and likely acceptance of) controlled hunting [71]. In Fiji, there is little available information on what the local perspectives on bats are and how this might affect their conservation. Known interactions between people and bats in Fiji include hunting and consumption, human–wildlife conflict around farming, bats roosting within human communities and interactions through cave tourism [57–60]. In Fiji, community engagement was key in securing the protection and ongoing management of Nakanacagi Cave, an invaluable maternal roost site of the Fijian mastiff/free-tailed bat (*Chaerephon bregullae*) [72,73]. Understanding people's current perceptions is therefore important for bat conservation and public health efforts, as perceptions influence how communities interpret risks, respond to conservation messaging and engage with initiatives such as citizen science, surveillance and wildlife stewardship.

This study aimed to investigate the perceptions of bats by people in Fiji and to quantify the reported frequency and types of interactions between bats and people. The goal of this research was to identify priority themes and approaches for education and conservation, as well as identify population groups that might be at higher risk of exposure to any zoonotic pathogens that may be present in Fijian bats through their interactions with bats in Fiji.

## 2. Material and methods

### 2.1. Ethics and permits

This study was approved by the Fijian Ministry of Education, Heritage and Arts (MOEHA) and included letters of support from the Ministry of iTaukei Affairs, Ministry of Health, Department of Environment, University of the South Pacific (USP) and NatureFiji-MareqetiViti (NFMV). MOEHA approved Human and Animal Ethics (RA 35/18).

Informed consent was obtained from participants prior to the interview using the specifically designed free, prior and informed consent (FPIC) form appendix 1.

### 2.2. Participants

Participation was restricted to adults aged 18 years or older. A total of 198 participants completed the survey. Quantitative responses (e.g. reported bat consumption) are presented descriptively as proportions within the sample. As the survey did not employ probability-based sampling, results should not be interpreted as population-representative estimates. The aim was to interview an even spread of men and women with varying roles within communities near bat roosting sites. Participants were either nominated by the village chief, self-volunteered or those who were participating at ‘grog time’; kava or grog, the national drink of Fiji, is traditionally served as part of a ceremonial atmosphere, most commonly in welcoming guests into a village and on important occasions.

### 2.3. Location

In accordance with the requirements of the Fijian Ministry of iTaukei Affairs, local guides were employed to assist the research group by liaising with village chiefs, translating interviews and facilitating transport. The three main islands (Viti Levu, Vanua Levu and Taveuni) were chosen owing to ease of access, time limitations and pre-existing contacts established by the two supporting organizations, USP and NFMV. Villages visited were selected owing to proximity to known bat roosting sites (published or local knowledge). In some locations, participants were visiting relatives or had married into the village. They answered the questions as if they were in their native/mother village, thereby broadening the survey distribution (figure 1). The fieldwork took place over a single seven-week period in August–October 2018.

**Figure 1.**
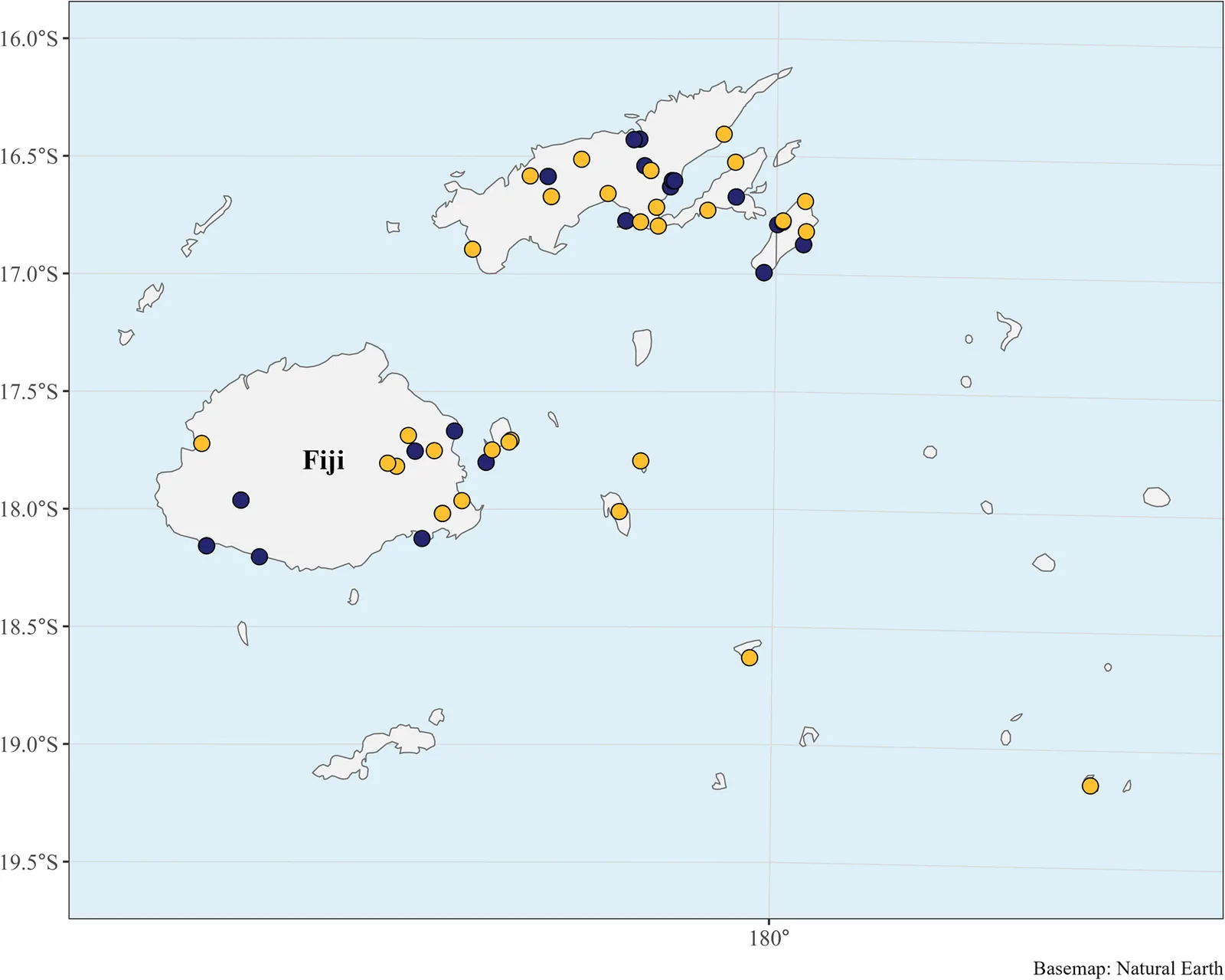
Map of interview locations. The physical location of the interviews (marked in blue) and the responders' home location if different to physical location (marked in yellow). Markers may represent one or more participants at a given location and are not scaled to sample size.

### 2.4. Survey design

The questionnaire was divided into two sections: the first to identify perceptions of bats (12 questions) and the second to identify interactions with bats (17 questions) (appendix 2). The combination of multiple-choice and open questions was asked during a 10–15 min face-to-face interview. Only 1 of the initial 35 participants elected to self-fill the printed questionnaire (available in both English and Bau Fijian) (appendix 3), so the remainder were transcribed at the time by the primary researcher (interviewer). Thirty-four per cent of interviews were voice-recorded as well as transcribed for cross-checking responses. In villages, the local guide was present for translating if necessary.

Participants were asked to provide their gender, age category, religion, region and education level to assess if these groupings are risk factors or predictors of behaviours. Information about an individual's self-reported status or role in the community was also collected. Responses were highly heterogeneous and inconsistently defined, precluding meaningful categorization or quantitative summary. As a result, these data were not analysed as a potential risk factor or reported in detail.

In this study, the term ‘perceptions’ is used descriptively to refer to participants' self-reported views, beliefs and evaluations of bats, as expressed through closed and open-ended questions. The survey was not designed to operationalize attitudes as a formal social-science construct or to distinguish between cognitive, affective or normative components.

### 2.5. Analysis

Here we provide qualitative and quantitative analyses. When presenting the quantitative results, the number of answers (the denominator) from the 198 participants varies when necessary. For example, some participants reported different answers for small insectivorous bats and large fruit bats (population trends, consumption, hunting and preparation); therefore, the total number of answers reported varies from 197 to 207. Each question was analysed in isolation with reference to the number of responses. The differences between ‘small’ and ‘big’ bats were not further distinguished in the analysis.

Quantitative results were tabulated in Microsoft Excel and analysed through R (v.3.5.2) [74] and plotted using ggplot [75]. We used logistic regression to model the risk factors for four different factors (response variables): (i) past and (ii) current consumption, (iii) contact and (iv) carcass preparation of bats using the glm function in the base R statistics package. The key covariates, age and education, were correlated, so age and gender were used as predictor variables with and without interactions, and the differences between the eight models (four response variables, with and without interactions) were tested using the Akaike information criterion (AIC). To assess whether risk perception influenced behaviour, recognition that bats can cause harm to humans was included as an additional predictor in all models. We assumed models were different if the AIC difference (ΔAIC) was greater than 2. Model results with coefficients, standard errors and *p*-values for the best models are in the appendix. Anonymous data and code to replicate these analyses are available at https://github.com/dtsh2/fiji_bats/.

Open-ended survey responses were reviewed using a descriptive thematic approach to identify recurring topics and illustrative examples relevant to the quantitative findings. Responses were read in full, key themes were identified through manual coding, and representative quotes were selected to provide context and support interpretation of the quantitative results. This process was exploratory and descriptive and was not intended as a formal theory-driven qualitative analysis.

#### 2.5.1. Reflexivity statement

This research team comprises researchers with backgrounds in veterinary science, ecology, epidemiology and wildlife health. One author (A.N.) is Fijian and serves as director of research and development within the Ministry of Forestry, Fiji, bringing local institutional knowledge and experience. The remaining authors are non-Fijian researchers. We acknowledge that researcher background, professional training and positionality may influence study design, data collection, interpretation and participant responses. We sought to mitigate these effects through collaboration with Fijian institutions, the use of local guides where required, informed consent procedures and transparent reporting of study limitations.

## 3. Results

Questionnaires were completed in full by 197 participants and 1 was partially completed (total *n* = 198). The interviews took place in 19 villages or towns with answers relating to 49 locations (figure 1). Men represented 54.5% (108/198) of participants, with the remaining 45.5% (90/198) women. The age distribution of participants is illustrated in figure 2 (supplementary material, appendix table S1).

**Figure 2.**
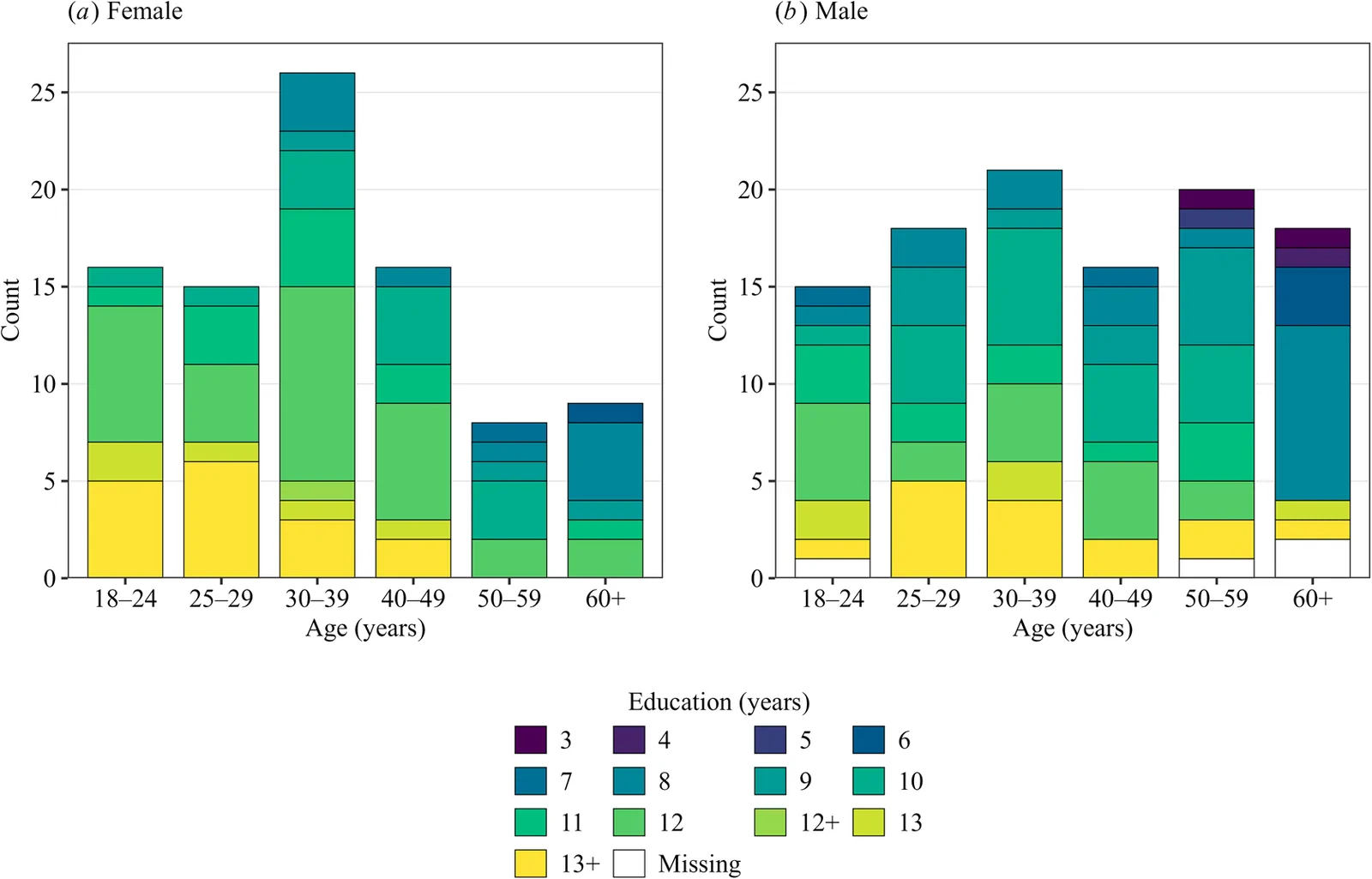
Age distribution, education in years (colours) and gender of participants.

The years of education of the study participants (figure 2) ranged from 3 years to 13+ years (participants who had completed, or were studying, a tertiary (e.g. college or university) qualification or trade were recorded as 13+ years). Women had more years of formal education, with 43.8% of women under 30 years of age having completed 13+ years of education, compared to 18.2% of men under 30 years of age (supplementary material, appendix table S2).

Participants reported a variety of religions, which were grouped as Catholic, Christian (denomination unspecified), Methodist, other Christian (e.g., Anglican, Pentecostal and Seventh-day Adventist), Hindu or no religion. Methodist was the most common religion (51.5%). Only six participants identified as a religion other than a Christian denomination (all six were Hindu) (supplementary material, appendix table S3). All six of these participants recorded no contact with bats, consumption or guano mining, but four out of six of them visited bat roost sites.

Reported roles ranged from formal positions (e.g. village leaders) to informal or multiple livelihoods (e.g. farming and fishing), often with overlapping or context-specific definitions.

### 3.1. Perceptions

#### 3.1.1. Bat numbers and types encountered

Participants were asked how many types of bats they had seen and frequently described the differences by colour and size. Local names were frequently used; however, this varied depending on location, and some names were used for different species depending on the area. For example, the name *beka lulu* on Taveuni described the Fijian flying fox (*Mirimiri acrodonta*), whereas on Vanua Levu this name described the Samoan flying fox (*Pteropus samoensis*) for some participants. The local guides had previously been involved in bat surveys, so species identification was clarified through known descriptive identifying traits. Two or more species of bat were seen by 55% (109/198) of participants in their area, commonly the Pacific flying fox (*P. tonganus*) and the Samoan flying fox. Bat populations were perceived to be increasing in the local area by 56.9% (115/202) of participants, decreasing by 21.8% (44/202), both increasing and decreasing by 6.4% (13/202) and 14.4% (29/202) of participants saw no change in population size/numbers. A single participant reported no bats locally in her area, so she did not comment.

Some participants referred to changes since Cyclone Winston in 2016 and/or suggested there was seasonal variation. One male participant aged over 60 on Taveuni commented:

*‘Before Winston thousands and thousands [of bats], after there is not as many—people ate them all as they lost their trees and breeding places’*.

Another male participant from the same village commented:

‘*[After] Cyclone Winston more than 1000's of bats were killed in different villages. (People were) not choosing by size or threatened status—[it was] more than 3 months before seeing another bat. Can see from edge of village the decrease. Count the bats flying over at nighttime and compare. Would talk about it at grog time—the decrease and what we can do about it. Most agree to stop cutting down big trees where they rest. If [I am] asked to give advice—please stop killing bats because they are very important and inform them the importance of bats, and show them the identifications’*. (Male, 60+ years old, Taveuni)

#### 3.1.2. Perceptions of bats as good versus bad

The participants generally had positive impressions of bats, with 63.1% (125/198) believing bats were good, 6.1% (12/198) saying bats were bad, 29.8% (59/198) saying they were both good and bad and 1% (2/198) expressing neither positive nor negative views.

Most participants who expressed positive perceptions of bats mentioned their use as a source of food (72.8%, 134/184). Bats are perceived to be clean, healthy and tasty. Other reasons included that bats are positive for farming and forests as they are responsible for seed dispersal and pollination (7.6%, 14/184), signal ripe fruit (4.9%, 9/184), provide guano as manure for gardens and crops (2.2%, 4/184) and can control insects (1.1%, 2/184). Bats were also perceived positively as they signal bad weather such as cyclones (4.3%, 8/184) and are harmless (2.2%, 4/184).

The main reason (71.8%, 51/71) for a negative perception of bats was that they eat the fruit, which was either destined for personal consumption or selling and therefore caused a loss of income. Other reasons included that bats were noisy at night-time (8.5%, 6/71), they cause harm and bite (5.6%, 4/71) and smell bad (4.2%, 3/71).

#### 3.1.3. Role and influence of bats

Responses to ‘What role do bats play/have in the environment?’ were open and subsequently categorized into the following groupings: no role, not sure, seed dispersal/replantation/pollination, weather, alarm, guano, source of food or other. Eleven respondents identified more than one role of bats in the environment, bringing the total number of responses to 211. Just over one quarter (27.5%, 58/211) of respondents felt that bats have no role in the environment. A similar number (27%, 57/211) identified that bats were involved in seed dispersal, replanting of fruit and forest trees, and pollination.

Further roles that were reported included acting as an indicator for changing weather, alarm for cyclones or alarm for waking up (9%, 19/211), providing guano/fertilizer to the environment (3.8%, 8/211) and providing a source of food for people (1.4%, 3/211). ‘Other’ roles were identified by 16.1% (34/211) of participants, including ‘clean up insects that try to eat the fruits’, ‘eat the fruits that humans waste or cannot reach’ and ‘attract tourism’.

Bats were perceived to have an influence (either positive or negative) on the day-to-day lives of 12.2% (24/197) of respondents. Positive reasons included a source of food, a source of income through tourism, insect control for their crops and dispersing seeds for new fruit trees. Negative influences included a loss of income from damage to fruits and a loss of sleep owing to noise at night.

#### 3.1.4. Bats as a cause of harm

Bats were reported to cause harm to humans through biting and/or scratching by 55.6% (110/198) of the participants. Two participants mentioned disease in other countries but not in Fiji. Another two participants commented that bats cause diseases, one stating they ‘cause diseases—when people eat the breadfruit that the bats have eaten then they get sick in the stomach’.

Of the 110 people who reportedly recognized bats can cause harm, 83.6% have been or are still in contact with bats. Consistent with this descriptive pattern, recognizing that bats can harm humans was a strong predictor of reporting contact with bats in adjusted logistic regression models (*p* < 0.001; supplementary material, appendix table S7). In contrast, recognition of harm was not associated with current bat consumption or preparation of bats as food (supplementary material, appendix tables S8 and S10).

#### 3.1.5. Who consumes bats as food

When asked who consumes bats, the majority (56.6%, 111/196) of participants stated that people in rural areas or villages were the ones who consumed bats, with 14 participants further detailing that bats were consumed only by poor Fijians in villages. Other responses by participants included 17.4% (34/196) reporting that only iTaukei Fijians consume bats, 11.2% (22/196) reporting that everyone consumes bats and 2.6% (5/196) reporting that only poor people ate bats (i.e. not only poor villagers). Other responses (12.2%, 24/196) included that ‘anyone’, ‘only some people’, ‘people in the highlands’/‘inland’ or on ‘remote islands’ ate bats. Two participants elected not to answer the question.

#### 3.1.6. Other perceptions regarding bat behaviour and diet

Along with the types of bats seen, their perceived population trends and the role of bats in the environment, participants were asked further questions regarding bat diet and activity. This was to further understand the level of knowledge of participants with respect to bat ecology and to understand further about local bat behaviour. These results are summarized in the appendix tables S4–S6 in the supplementary material.

### 3.2. Interactions with bats

#### 3.2.1. Contact with bats and injury from bats

Contact with blood, saliva, urine or faeces from a bat was reported by 51% (101/198) of participants. However, 64 participants who answered ‘no’ to the initial survey question on direct contact with bats (defined as touching or handling a bat) subsequently reported having bat-related contact in additional questions, including injury from bats, catching, preparing, and/or consuming bats, mining/using guano and/or using bats for other uses. This increased the total to 83.3% of participants having had contact with bats. Injuries, either through being bitten or scratched by a bat, were reported by 29.8% (59/198) of participants. Statistically, age, gender and recognition that bats can harm humans were predictors of bat contact. Older participants (40–49 and 60+ years old, *p =* 0.01 and <0.001, respectively), males (*p* < 0.001) (figure 3), and those recognizing bats can cause harm (*p* < 0.001) were more likely to report contact with bats (supplementary material, appendix table S7).

**Figure 3.**
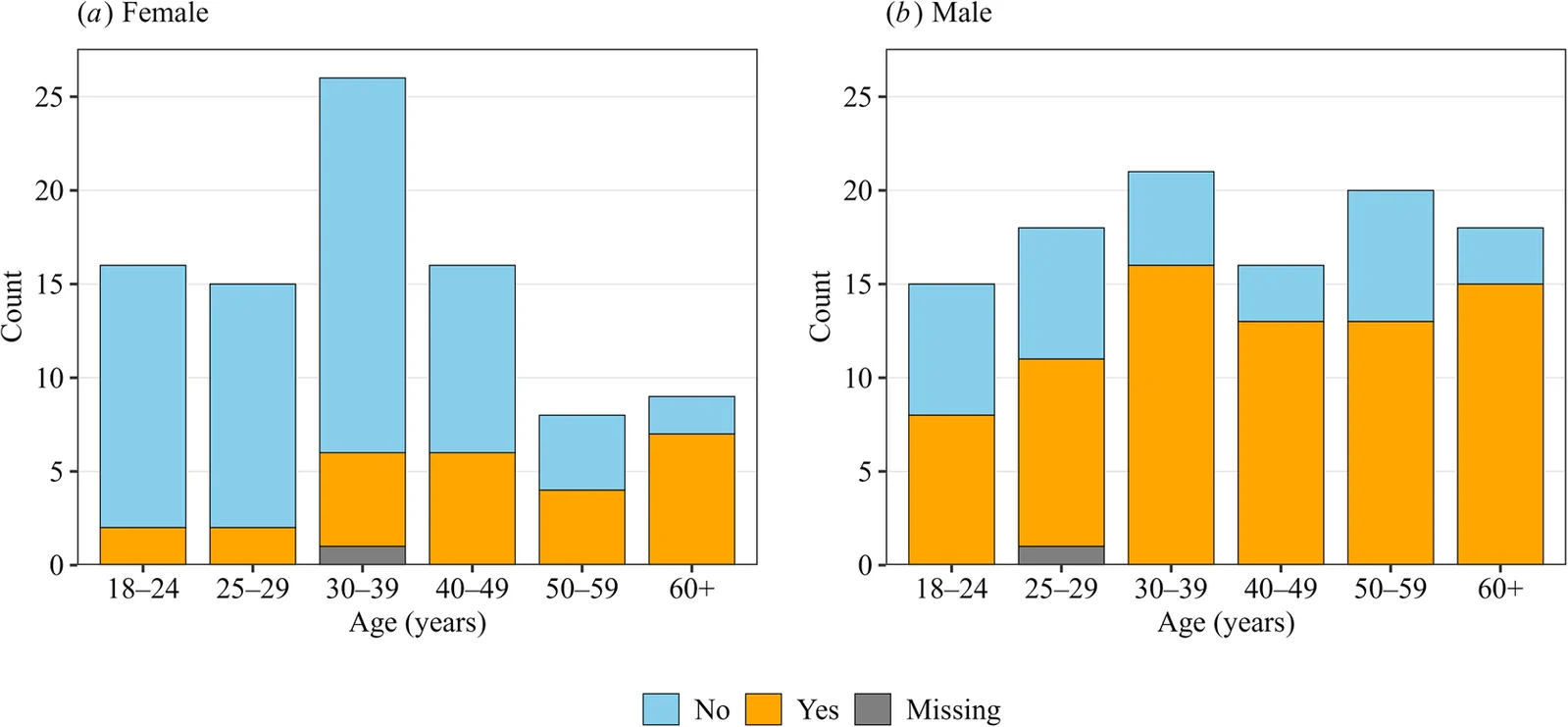
Reported human contact with bats in Fiji, categorized by age and gender.

#### 3.2.2. Consumption of bats as food

Participants were asked if they had consumed bat meat in the past and if they currently consumed bat meat. If the answer was yes, then the quantity, frequency, source and how the meat was prepared were recorded. In the past, 79.7% (161/202) of participants consumed bat meat. Bats were currently consumed by 27.5% (56/204) of respondents, 24% (49/204) of respondents will continue to eat bats, and 48.5% (99/204) do not currently consume bat meat. Age and gender are predictors of current bat-eating habits; specifically, men are more likely to eat bats than women are (*p =* 0.01), and respondents aged 40 years and older are more likely to eat bats than 18–24-year-old respondents (*p-*values range from 0.03 to 0.004) (figure 3; supplementary material, appendix table S8). Recognition that bats can cause harm was not associated with current bat consumption (*p* = 0.72).

However, these figures have fallen from 161/202 participants who said they had eaten them in the past (figure 4). Among those reporting past consumption, males were significantly more likely to report having eaten bats (*p* = 0.008; supplementary material, appendix table S9). Recognition that bats can harm humans showed a borderline positive association with past bat consumption (*p* = 0.06), while age effects were not statistically significant. For past bat consumption, models including age–gender interaction terms provided a better fit than main-effects-only models (ΔAIC > 2).

**Figure 4.**
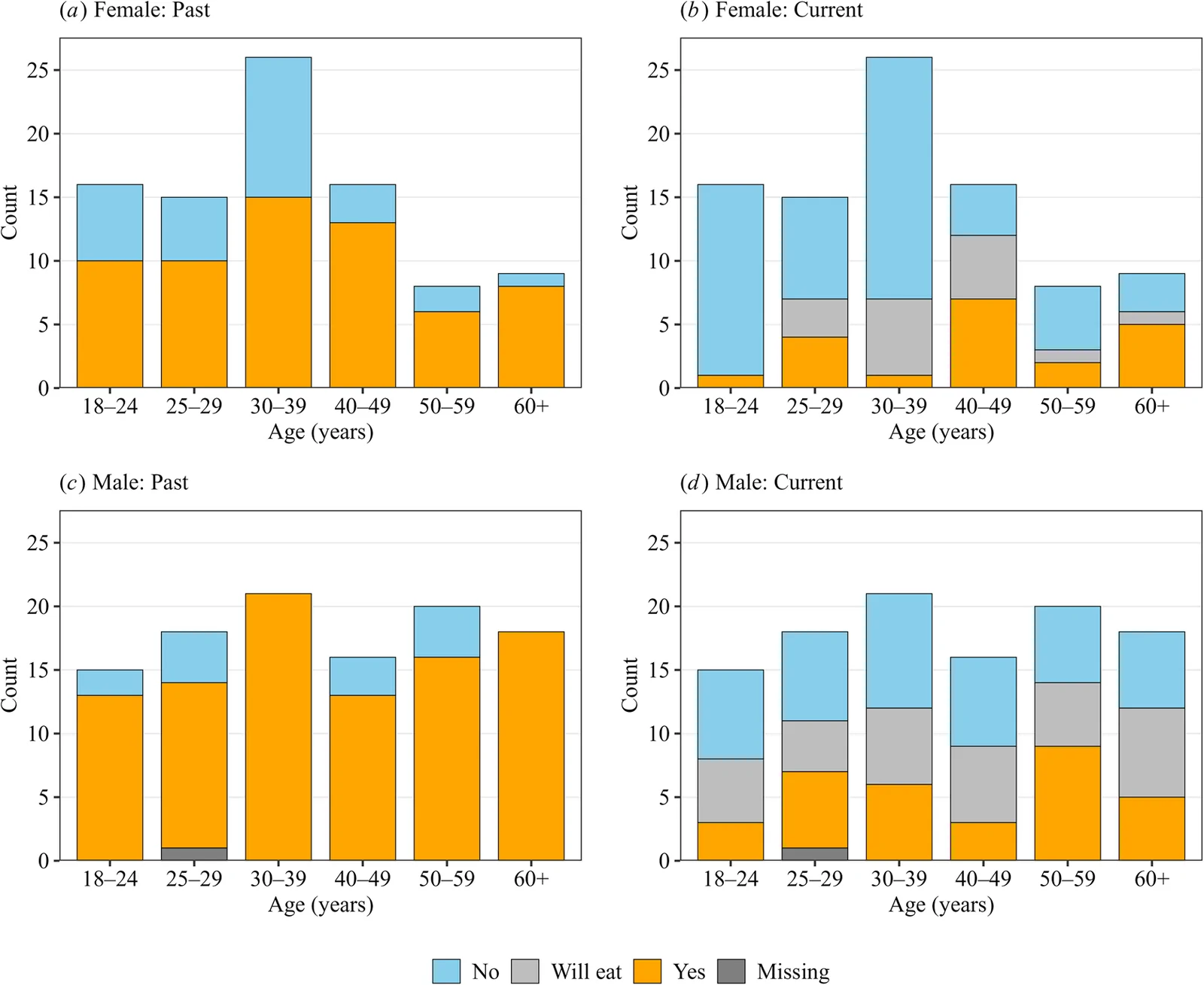
Bat consumption by people in Fiji, categorized by past and present consumption habits, gender and age cohort.

In one village located near a large limestone cave that supports a roost of Fijian blossom bats (*Notopteris macdonaldi*), bat consumption was reported to be more common among those of higher status, the majority of whom are men.

Bats seem to be an important (and favoured) seasonal protein source to many communities:

*‘In my village most people like to eat bats. Everyone is happy when someone catches one at night because it means they will have meat for the night. Other source of meat is wild pigs once every 2 weeks.’* (Male, 30–39, East Viti Levu)

*‘Clean as only eat fruit. Eaten by poor people—kind of chicken so is a free chicken being caught in the night.’* (Male, 40–49, Taveuni)

*‘It is free meat unlike chicken, beef, etc. Very clean meat. Can't buy it*.*’* (Female, 30–39, West Viti Levu)

#### 3.2.3. Other reported uses of bats

Infrequently reported other uses were medicinal to alleviate/treat pain, wounds, diabetes, blood pressure, and heart, eye and gastrointestinal ailments. It was also commented that bats are sold to Chinese tourists for traditional medicine.

Non-medicinal uses/symbols of bats reported included making jewellery from the jawbone and the use of bats as a family/village emblem. Some people reported a belief that ‘if a bat is seen flying during day and making noise, then someone is about to die’ (female, 25–29, West Viti Levu).

#### 3.2.4. Hunting of bats

Almost two-thirds of participants (62.8%, 130/207) had caught bats in the past. When asked if they were currently hunting, 79% reported no; 14.6% of people were currently active in bat hunting, with the remaining 6.4% of participants recording that they intend to hunt bats in the future. Of the 37.2% (77/207) responders who reported never catching a bat, four individuals responded that they would go along with the hunting party for fun.

Catching and consumption of insectivorous bats has changed in one village on Vanua Levu since the nearby cave became protected, as it is the only known maternal colony of the Fijian mastiff/free-tailed bat (*C. bregullae*).

#### 3.2.5. Preparation of bats as food

A majority of responders (65.5%, 131/200) reported being involved in preparing bats as food. In our statistical analysis, the model with age and gender (AIC = 239) was a better model (ΔAIC > 2) than that with interactions (AIC = 245). Age was a predictor of who prepared bats as food, with older people in three age classes (30–39, *p* = 0.02; 40–49, *p* = 0.007; ≥60 years, *p* < 0.001) more likely to report being involved in preparing bats as food than 18–24-year-olds (supplementary material, appendix table S10). Recognition that bats can cause harm was not associated with preparation of bats as food (*p* = 0.21).

In general, participants reported preparing bats weekly during bat season, boiling approximately one to five large fruit bats at a time. Some participants reported cooking between 30 and 100 per ‘season’ (summer fruit season was referred to as ‘bat season’). Either the pot size or number of bats caught was reported to be the determining factor as to how many bats would be prepared at a time. The bats were generally deceased prior to cooking; however, they were not always dead when brought back to the village. Some participants reported being bitten or scratched when catching the bats out of bags to cook them.

Up to 200 Fijian blossom bats (*N. macdonaldi*) would be cooked at one time in one village, but only once a year. In another village, the Fijian mastiff/free-tailed bats (*C. bregullae*) were no longer allowed to be harvested. Historically, a household would cook up to 50 of these species at a time when harvested (approx. twice a year).

#### 3.2.6. Use of bat guano as garden fertilizer

Collecting guano for fertilizer was reported as a current activity by 6.6% (13/196) of people, whereas 7.7% (15/196) reported collecting in the past (11/13 current participants used guano in the past and currently). The additional two people who did not historically collect guano had either moved into an area where guano is commonly used or had recently learned about its use as a fertilizer.

Residents near Wailotua cave report that in the 1930–1940s there was commercial (Mr Lauchardt) guano mining in Wailotua cave, with old tracks and electrical wiring from lighting still remaining in the cave. Residents reported still collecting approximately 10–25 kg of guano for fertilizer one to two times a year for personal use. One participant used to sell the guano to nearby villages but no longer does this.

Guano is still collected/mined from the cave in Nakanacagi. On average, it is collected once a year, with amounts varying from as little as 1 × 10 kg bag up to 20 × 25 kg bags. Most participants responded that it was only for personal use, but two people also reported selling bags of guano for FJD$7–10/bag (bag size/weight not specified).

#### 3.2.7. Visits to bat roosting sites

Visiting bat roosts (trees or caves) was reported by 57.4% (113/197) of responders; reasons for the visits included work, interest/recreation and hunting or because the bats lived around their house.

#### 3.2.8. Actions on finding a sick or injured bat

Participants reported that if they found a sick or injured bat, they would either kill and eat the bat (28.4%, 56/197), leave it alone (25.4%, 56/197), care for it (12.2%, 24/197) or kill it to give away to someone to eat or bury/cremate the bat (9.6%, 19/197). Other answers included moving the animal, and either killing, caring for or leaving it alone depending on how the bat looked. A few participants noted that they would not know if it was sick or not.

#### 3.2.9. Current bat education and conservation

Participants were asked if they had any further comments regarding bats at the end of the questionnaire. Most reiterated their previous answers and comments that bats were healthy, clean animals that were good for eating. Some participants, especially males, expressed interest in learning more about bats, their importance and conservation efforts:

‘*More information for people to preserve how they breed to protect them so they can multiply*.’ (Male, age 50–59 years, Taveuni)

*‘Bats are getting more precious now and we should be more educated. We need to decrease development and cutting down trees as the lower soil needs more nutrient/fertiliser from bat poo.’* (Male, age 60+ years, Taveuni)

‘*Only known about it for eating. It would be good to have more education*.’ (Male, age 40–49 years, Taveuni)

NFMV had started outreach and education programmes prior to this study:

‘*NGOs have come to do workshops to increase education about bats*.’ (Male, age 50–59, Taveuni)

Anecdotally, respondents appeared to be more aware and educated regarding the role of bats in the environment compared to those who had not participated in these programmes. Further research would need to be conducted to fully review the effectiveness of such education programmes.

## 4. Discussion

This survey aimed to understand the perceptions of Fijian people about bats and to quantify the type and frequency of any interactions reported between bats and people and to identify priority issues that may be affecting community engagement in bat conservation in Fiji. A second objective was to identify potential points of zoonotic disease exposure between bats and people in Fiji. The results of this study show a high frequency and variety of interactions between bats and people in Fiji. This presents numerous avenues for exposure to zoonotic pathogen spillover, including the use of bats as food and bat guano as garden fertilizer. There appear to be generational and contextual differences in how bats are perceived, with older participants more likely to describe past practices and higher levels of bat use and younger participants more frequently emphasizing ecological roles and reduced consumption.

Engaging communities in protecting key roosting sites, bat food sources and sustainably harvesting bats (and encouraging decreased consumption) are key goals in working towards bat and habitat conservation in Fiji. Community discussions could include education about, and protection of, bats in Fiji while acknowledging the dietary role that these wildlife species play and ideally addressing the availability and capacity to obtain potential dietary alternatives. As well as community science [65] and environmental education programmes [63], the conservation strategy used for locally managed marine areas already in place in Fiji [76] could guide strategies on land. In the case of marine areas, Kim *et al* [76] investigated the socio-economic factors affecting the sustainable use of natural resources in rural communities in Fiji. Key dynamics that were focused on included the community's trust in external partners (government and NGOs), individual participation in decision-making regarding natural resource management, motivations behind management practices and rules in the community (with respect to fishing) and whether fishing was a source of income and/or protein. While bats do not appear to have a direct source of income for most participants in this study, there was widespread consumption of bat meat, typically as a seasonal delicacy, yet also a dependable, free and accessible source of protein. Future resource management, land protection and community engagement could combine this existing framework with the new understanding of the complex human–bat interactions and perceptions highlighted in this study. Specifically, conservation planning could integrate participatory approaches that build trust with local communities, incorporate traditional knowledge and address both ecological and livelihood needs. For example, land use policies could support the protection of key bat habitats near villages while promoting alternative protein sources or livelihood strategies that reduce hunting pressure, accompanied by targeted education campaigns to shift risk perceptions around bats and zoonoses.

Contrary to previous bat population surveys in Fiji [60,77], many participants perceived that bat population trends overall were increasing. However, comments from older participants indicated that the bat populations may have decreased, as bats are no longer found hanging in large chains from roosting trees. Comments were also made that it is harder to find bats or catch bats now than it used to be. There are a number of possible explanations as to why more bats are being seen, including the movement of bats into populated areas owing to habitat encroachment and deforestation [78,79]. These factors may have led to decreased native food sources, meaning that bats are switching to agroforests or crops that are easier to access [80]. A population decrease was also noticed since the most recent category 5 cyclone (Cyclone Winston, 2016) at the time of the study. Other studies have monitored populations following large cyclonic storms and found that the abundance and behaviour of bat species varied with the impact on primary forest, secondary forest and species ecology, as well as short-term displacement versus long-term population counts [81–85]. Fijian bat populations were not formally monitored after this event, but very high levels of bat consumption post Cyclone Winston were reported from villagers in south Taveuni. Perceptions of bat population trends are important because they may influence conservation priorities. If bat populations are perceived to be increasing, activities that are detrimental to bats – such as hunting, deforestation and cave tourism – may be viewed as less urgent to address. Further work is needed to actively monitor Fijian bat population trends.

With the majority of participants perceiving bats as either good or both good and bad, this perspective could be reinforced and used as a focus for bat conservation. Reasons for bats being seen as good included their importance in the environment and their suitability as a food source. The majority of plant species serviced by bat species in Fiji are valued by humans for economic, medicinal and cultural uses [50], which is an important link to include in community education. The role of bats as pollinators and seed dispersers was recognized by some individuals in some villages, including where previous community engagement, education and research had been conducted about bats. While this may reflect prior exposure to information about bats, this study did not assess the presence or impact of specific education or engagement programmes and, therefore, cannot attribute these perceptions to previous interventions or link them to conservation behaviours. However, this suggests that previous education efforts could have increased knowledge in this area specifically about bats; however, it did not necessarily translate to any proactive conservation efforts. Trewhella *et al*. [63] and Hoffmaster & Vonk [86] also highlight the importance of education about the role of bats in the environment and how bats can benefit people, which may increase the overall positive perception of bats.

Bats are often perceived negatively for either being directly harmful to humans or indirectly through damage to fruit crops that results in loss of income [67,68,87,88]. In this study, just over half of the participants reported that bats can harm humans through biting or scratching. Even though people recognized that bats can cause harm, the majority of these participants (approx. 83%) have been or still are having contact with bats, and recognition of harm was positively associated with reporting bat contact but was not associated with reduced bat consumption or preparation behaviours. Similar behaviours were also reported in Ghana, where despite the risk of contracting viruses, such as Ebola virus and other bat-borne viruses, bats were still being caught, sold and consumed [89]. The consumption of fruit crops by foraging bats was a main reason why people perceived bats to be negative. Income loss was not investigated in this study, nor was it mentioned as a direct effect from the bats by the participants.

It is important to encourage positive perceptions when highlighting the benefits of bats and working towards the conservation of a species; bats can, however, pose a potential risk of disease to humans [90]. This study has highlighted an interesting conundrum for conservationists, where part of the positive perception of the Fijian bats is that they are seen as a ‘fresh and clean’ food source. Peel *et al.* [91] also investigate bat–human interactions and hunting, including isolated island populations, in Africa, and similarly note beliefs about how ‘clean’ bat meat is. Conservationists might discourage consumption of the bats by educating people about the zoonotic disease risks associated with bat contact and consumption; however, this approach risks tainting people's perception of the bats and may lead to increased pressure on roosts and foraging habitat. The negative effect on bat conservation associated with a fear of zoonotic disease has been demonstrated in Australia in relation to Hendra virus, where bat roosts have been deliberately destroyed to reduce perceived disease risks around horse stables and paddocks [9,92,93]. In Kenya, bat roosts have also actively been disturbed or bats killed owing to negative perceptions surrounding bats, retaliation to crop damage, or myths and beliefs about bats and disease [68]. Tragically, culling of Marburg virus (a relative of Ebola virus) hosts, *Rousettus aegyptiacus*, in Uganda has previously been seen to have likely contributed to a resurgence of the virus in bat colonies and subsequent fatal human infection [94]. Bats perform important ecosystem services, and both depend on the native habitats and support ecosystem health, so with habitat loss being a mechanism that can increase the risk of pathogen spillover from bats and other species through changing infection dynamics and contact patterns [95–98], maintaining healthy habitat for bats, including land management and sustainable farming practices, can play an important part in both conservation and disease prevention strategies, and maintaining ecological integrity as an important countermeasure to infectious disease emergence is required [95,98]. As some participants made positive statements about preserving trees, and >60% of participants had positive impressions of bats, this seems a positive avenue for future work in Fiji.

Zoonotic diseases from animals may be spread via direct contact with saliva, blood, urine, faeces or birth products, or indirect routes through arthropod vectors [8]. This survey asked participants about direct contact with bat saliva, blood, urine and faeces, as well as identifying other opportunities for exposure—hunting, consumption, guano mining and visiting roost sites. Some participants also reported other forms of contact such as medicine and jewellery. These routes have previously been reported [60,77], however never quantified or explored, which is especially useful in determining who is at risk of exposure to a zoonotic disease, if present, in bats in Fiji. This study identified that age, sex and recognition that bats can cause harm are predictors of contact with bats, with older people, males and those recognizing harm more likely to have contact with bats. With regard to the preparation of bat meat—older people are more likely to prepare bat meat than younger people, especially people aged between 40 and 49 and those over 60 years of age, potentially putting these age groups at greater risk. Our study, therefore, identifies pathways that might lead to infection and the people at risk, and together these could be targets for promoting the adaptation of wearing personal protective equipment, such as gloves and masks, with appropriate disposal, and other recommendations for guano harvesting from the IUCN Bat Specialist Group that will protect people from exposure [99,100].

Interpretation of reported changes in bat consumption over time, like other responses, should be treated with caution. Historical consumption was based on participants' retrospective self-reports, which may be subject to recall bias, nostalgia effects or changing social norms around hunting and consumption. As such, differences between reported past and current bat consumption should not be interpreted as evidence of a true temporal decline, but rather as an indication of how participants perceive changes in their own behaviour over time. Here, consumption of bat meat in Fijian villages appears to be common, with more than 79% of participants reporting having eaten bat in the past. This number decreased to 51.5% of participants when identifying those currently consuming or will consume bat meat in the near future. These findings are similar to bat consumption in Ghana [89] and Thailand [66]. Recognition of bats as a potential source of harm did not appear to deter current bat consumption or preparation practices, highlighting a disconnect between risk awareness and behaviour. Age and gender are predictors of current bat consumption, with men more likely to eat bats than women as well as people aged over 40 years of age. Age and education were correlated in this sample, and education was therefore not included as an independent predictor of bat consumption in statistical analyses. A lower proportion of participants reported current bat consumption or hunting compared with retrospective self-reports of past behaviour. However, these comparisons rely on recall of historical practices and should not be interpreted as evidence of a true temporal decline. Participants attributed perceived changes to factors such as bats being harder to hunt, increased awareness of bats' ecological importance, legal protection of certain roosts, dietary changes and migration away from villages; however, this study was not designed to evaluate the relative importance or causal role of these factors. We did not specifically ask about the hunted species, but references to them being clean because they ate fruit and summer fruit season being referred to as ‘bat season’, along with their larger size, would suggest larger, frugivorous bats are preferred.

Unlike in some countries [48,101], bats are not readily sold in markets in Fiji but rather caught for personal or village consumption. Consumption of bat meat was generally reported as seasonal and associated with positive comments of being ‘tasty, clean and healthy’, but it may also be an important source of protein for some villages. The demographics in this study were not comprehensive enough to draw conclusions on whether rural versus urban or coastal versus inland inhabitants were more or less likely to consume bat meat. Understanding the traditional uses and ongoing importance of bats as bushmeat is important to navigate the sensitive topic of decreasing consumption to conserve bat populations [48,102]. It is also possible that religion may be an indicator of exposure risk to bats. This may also be true of ethnicity, for although ethnicity was not a specific survey question, only six Hindu participants, who also identified as Indo-Fijian, participated; the majority of participants were iTaukei Fijian. With so few Hindu participants, this observation needs to be interpreted with care.

The main limitation in this study was sampling/participant bias. Participants were either nominated by a village chief (possibly a subsection of the village was therefore targeted), volunteered (and were more willing to elaborate on answers or provide additional information) or were the vendors in markets free to talk at the time rather than a precalculated subsection of the population. Participation was also limited to adults, and therefore, no conclusions can be made regarding the perceptions and interactions of bats and minors or differences in risk factors. Most interviews took place in villages close to bat roosting sites or marketplaces with fruit and vegetable vendors; therefore, there was already a potential bias towards bat interactions. It can be argued, though, that as this study was designed to quantify these interactions, focusing time and resources on communities that were more likely to be exposed to bats provided an in-depth understanding. There were a few interviews where translation was required for the majority of the interview, and it cannot be confirmed if the questions were asked consistently or if respondents were led to their answers. Other limitations included the length of the study period, personnel support for physically collecting data, financial constraints, transport limitations and permission delays. Despite limitations and challenges, this pilot study provides the first quantitative and qualitative study of interactions between people and bats in Fiji, with interviews having been undertaken across a significant part of the country (figure 1), leading to the identification of risk factors for bat contact, consumption and preparation, as well as highlighting areas for potential bat conservation interventions and measures.

The importance of understanding the level of knowledge about a focal species for conservation efforts is vital for ensuring that these efforts are efficient and effective [4,5,63,79]. Although participants were asked to comment on population trends, this study did not cover or monitor the populations of the six species present in Fiji. Baseline data about the distribution of the six species of bats in Fiji have been gathered by Palmeirim *et al*. [77] and Scanlon *et al*. [60]; however, there is an opportunity for community engagement and citizen science for ongoing monitoring of population trends, activity and diet.

In summary, through face-to-face questionnaires, this study identified complex human–bat relationships and interactions, determined that potential at-risk groups for exposure to zoonotic disease from bats are present and highlighted areas for targeted education efforts towards bats and conservation. Overall, the perception of bats in Fiji by participants in this study was largely positive, but the persistent use of bats as food creates a difficult conundrum for bat conservation. This positive attitude can be used for future education and conservation efforts, but if zoonotic diseases are present in Fijian bats, it will be important to consider how this is reported to the wider community to avoid creating negative perceptions of bats, as is the case in many countries. If Fijian bats carry zoonotic pathogens, this study has identified many potential routes of exposure of Fijian people to zoonotic disease. Highlighting how disease is spread, how to decrease possible exposure as well as the benefits of bats to the community and environment are key messages to safeguard public health and improve bat conservation in Fiji.

## Supplementary Material



## Data Availability

Data and relevant code for this research work are stored in GitHub (https://github.com/dtsh2/fiji_bats) and have been archived within the Zenodo repository [103]. Supplementary material is available online [104].
