# Supplementary material for: People and bats in Fiji: perceptions and interactions
Source: R Soc Open Sci. 2026 Nov 4;13(11):250383. doi: 10.1098/rsos.250383 (PMC13730655; doi:10.1098/rsos.250383)
Supplement: Supplementary file 1 [file rsos.250383.zip › rsos.250383/rsos250383_si_001.docx]

*Appendix 1: Free and Prior Informed Consent Fijian Bat Research*

*Appendix 2: Questionnaire - Fijian bats: interactions with people and disease surveillance focusing on zoonotic pathogens - English*

**Fijian bats: interactions with people and disease surveillance focusing on zoonotic pathogens**

**QUESTIONNAIRE**

**Subject details** *Questions 1 – 7*

*Please note your name and address is only recorded in case we require further information from you while in your region. It is not recorded next to your responses in the research database or disclosed to third parties.*

1. Name: ________________________________________________________ (optional)
2. Address:_______________________________________________________ (optional)
3. Region: _______________________________________________________________
4. Age:
   a. 18-24
   b. 25-30
   c. 30-39
   d. 40-49
   e. 50-59
   f. 60+
   g. Not specified
5. Gender:
   a. Male
   b. Female
   c. Other
6. Role in the community (if applicable): _____________________________

**_____________________________**

7. Education: _________________________

**Perception of bats** *Questions 8 - 19*

*Please answer the following questions with your own personal experiences in mind. Please feel free to add any extra comments with your answer to the questions. Please state if you do not wish to answer the question asked. With your permission the following interview will be recorded with a voice recorder. The recording will not be made public. It is only for record keeping and to assist with ensuring the answers are recorded correctly.*

8. Do you see bats near where you live or work?
   a. Yes
   b. No
9. How many species or types of bats do you see? ________________________________

Comments _____________________________

10. What do you think bats eat?
    a. Fruit
    b. Seed
    c. Plants
    d. Insects
    e. Other _______________
11. What have you seen bats eat?
    a. Fruit
    b. Seed
    c. Plants
    d. Insects
    e. Other _______________
12. At what times do you see bats being active (eg. flight, eating, vocalising)?
    a. Dawn/sunrise
    b. Morning
    c. Afternoon
    d. Dusk/sunset
    e. Night
    f. Other _______________
13. In your experience, have the number of bats you have observed –
    a. Decreased?
    b. Increased?
    c. OR Stayed the same? - G*o to question 15*
14. Why do you think the numbers of bats have changed?
    a. Increased - __________________________________________________________
    b. Decreased - __________________________________________________________
15. In your opinion, are the bats -
    a. Good - Why? __________________________________________________________
    b. Bad - Why? __________________________________________________________
    c. Both – Why (unless answered both above)? _____________________________
    d. Neither good nor bad. _____________________________
16. What role do bats play in the wider environment? _________________________________
17. Do the bats influence your day to day life?
    a. Yes – How? _____________________________
    b. No
18. Can bats harm humans?
    a. Yes – How? _____________________________
    b. No – Why? _____________________________
19. Who/which people do you think eats bats? Eg. Poor, wealthy, urban, rural, status, everyone? ______________________________

**Interactions with bats** *Questions 20 - 36*

*The following questions are to identify the different interactions between people and bats by asking you about your personal experiences both past and present. There may be some questions that are repeated from the previous section. There will also be some questions about the different uses of bats. Please feel free to add any extra comments with your answer to the questions. Please state if you do not wish to answer the question asked.*

21. Are there any bats roosting near where you live, commute or work?
    a. No	*- Go to question 22.*
    b. Yes
22. Where do they roost/sleep?
    a. Arboreal (sleep in trees)?
    b. Caves?
    c. Man-made infrastructure (eg. houses, bridges)?
    d. Other ___________________________
23. Have you ever been in contact with blood, saliva, urine or faeces from a bat?
a. No
b. Yes _____________________________
24. Have you ever been injured by a bat? (eg. Scratched or bitten?)
a. No
b. Yes _____________________________
25. In the **PAST** have you eaten bat meat?
    a. No	*- Go to question 25.*
    b. Yes
    c. How many/much? _____________________________
    d. How often?
       d.i. Daily
       d.ii. Weekly
       d.iii. Monthly
       d.iv. Every 3 months
       d.v. Every 6 months
       d.vi. Once a year
       d.vii. Other__________________
    e. Where did you source the products from?
       e.i. Self-caught
       e.ii. Village caught
       e.iii. Market
       e.iv. Other ___________
    f. How were they prepared? _____________________________
26. Do you **CURRENTLY** eat bat meat?
    a. No	*- Go to question 26.*
    b. Yes
    c. How many/much? _____________________________
    d. How often?
       d.i. Daily
       d.ii. Weekly
       d.iii. Monthly
       d.iv. Every 3 months
       d.v. Every 6 months
       d.vi. Once a year
       d.vii. Other___________________
    e. Where did you source the products from?
       e.i. Self-caught
       e.ii. Village caught
       e.iii. Market
       e.iv. Other _________________
    f. How are they prepared? _____________________________
27. In the **PAST** have you used bats for anything **other than** meat?
    a. No	*- Go to question 27.*
    b. Yes – please specify ___________________________
    c. How many/much? ___________________________
    d. How often?
       d.i. Daily
       d.ii. Weekly
       d.iii. Monthly
       d.iv. Every 3 months
       d.v. Every 6 months
       d.vi. Once a year
       d.vii. Other__________
    e. Where did you source the products from?
       e.i. Self-caught
       e.ii. Village caught
       e.iii. Market
       e.iv. Other __________
    f. How were they prepared? ___________________________
28. Do you **CURRENTLY** use bats for anything **other than** meat?
    a. No	*- Go to question 28.*
    b. Yes – please specify _________________
    c. How many/much? ___________________________
    d. How often?
       d.i. Daily
       d.ii. Weekly
       d.iii. Monthly
       d.iv. Every 3 months
       d.v. Every 6 months
       d.vi. Once a year
       d.vii. Other__________
    e. Where did you source the products from?
       e.i. Self-caught
       e.ii. Village caught
       e.iii. Market
       e.iv. Other ______________
    f. How were they prepared? ___________________________
29. Have you personally caught bats in the **PAST**?
    a. No	*- Go to question 29.*
    b. Yes	Year of last catch ___________________________
    c. Did you actively hunt (and how) OR find them by chance?____________________
    d. How many? _________________________
    e. How often?
       e.i. Daily
       e.ii. Weekly
       e.iii. Monthly
       e.iv. Every 3 months
       e.v. Every 6 months
       e.vi. Once a year
       e.vii. Other__________
    f. Purpose?
       f.i. Consumption
       f.ii. Income
       f.iii. Gift
       f.iv. Other _________
30. Do you **CURRENTLY** catch bats?
    a. No	*- Go to question 30.*
    b. Yes
    c. Are these actively hunted (and how) OR find them by chance?_________________
    d. How many? ________________________
    e. How often?
       e.i. Daily
       e.ii. Weekly
       e.iii. Monthly
       e.iv. Every 3 months
       e.v. Every 6 months
       e.vi. Once a year
       e.vii. Other__________
    f. Purpose?
       f.i. Consumption
       f.ii. Income
       f.iii. Gift
       f.iv. Other _____________
31. Do you prepare bats to be consumed for eating?
    a. No	- *Go to question 31*
    b. Yes
    c. How many? ________________
    d. How often?
       d.i. Daily
       d.ii. Weekly
       d.iii. Monthly
       d.iv. Every 3 months
       d.v. Every 6 months
       d.vi. Once a year
       d.vii. Other___________
    e. How are the bats prepared for eating? ____________________________

**Guano**	*Questions 31 – 32*

32. Have you used/mined guano in the **PAST**?
    a. No	*- Go to question 32.*
    b. Yes
    c. How much? ___________
    d. How often?
       d.i. Daily
       d.ii. Weekly
       d.iii. Monthly
       d.iv. Every 3 months
       d.v. Every 6 months
       d.vi. Once a year
       d.vii. Other___________
    e. Source?
       e.i. Self-mined
       e.ii. Purchased
       e.iii. Other _______________
    f. Purpose?
       f.i. Personal use
       f.ii. Income
       f.iii. Gift
       f.iv. Other ______________
33. Do you **CURRENTLY** use/mine guano?
    a. Yes
    b. No	*- Go to question 33.*
    c. How much? ___________
    d. How often?
       d.i. Daily
       d.ii. Weekly
       d.iii. Monthly
       d.iv. Every 3 months
       d.v. Every 6 months
       d.vi. Once a year
       d.vii. Other__________
    e. Source?
       e.i. Self-mined
       e.ii. Purchased
       e.iii. Other __________

Purpose?

e.iv. Personal use
e.v. Income
e.vi. Gift
e.vii. Other __________

**Roosting sites**	*Questions 33 - 36*

34. Do you visit roosting sites?
    a. Yes
    b. No	*- Go to question 34.*
    c. Where is the roosting site? eg. Tree, cave, building ___________________________
    d. Purpose? ___________________
    e. How often?
       e.i. Daily
       e.ii. Weekly
       e.iii. Monthly
       e.iv. Every 3 months
       e.v. Every 6 months
       e.vi. Once a year
       e.vii. Other__________
35. If you see an injured or sick bat – what do you do?
    a. Leave it alone
    b. Move it
    c. Care for it
    d. Kill it
    e. Sell it
    f. Eat it
    g. Other ____________________________
36. Do you have any other interactions with bats? Yes or No
    a. Please specify __________________________________________________________
37. Are there any further comments you would like to add?
    _______________________________________________________________________________________________________________________________________________________________________________________________________________________________________

Thank you 😊

*Appendix 3: Questionnaire - Fijian Bats: interactions with people and disease surveillance focusing on zoonotic pathogens - Fijian*

**Fijian bats: interactions with people and disease surveillance focusing on zoonotic pathogens**

**QUESTIONNAIRE**

**Ai Vakatataro ni vakadidike ni Beka(bats) I Viti**

**Subject details** *Questions 1 – 7*

*Please note your name and address is only recorded in case we require further information from you while in your region. It is not recorded next to your responses in the research database or disclosed to third parties.*

**Nomu I Tukutuku**

1. Yacamu	________________________________________________ (optional)
2. Nomu I Tikotiko	________________________________________________ (optional)
3. Nomu yasana	________________________________________________ (region)
4. Nomu Yabaki :
   a. 18-24
   b. 25-29
   c. 30-39
   d. 40-49
   e. 50-59
   f. 60+
   g. Not specified
5. Toqa ga e dua:
   a. Tagane
   b. Yalewa
6. Na nomu I tutu vakavanua ena nomu I tikotiko se koro
   _______________________________________________
7. Vakatagedegede ni Vuli (education)

_____________________________

**Ai vakatakilakila ni Beka**	Questions 8 - 19

*Sa kerei mo ni qai sauma na taro me veidonui kei na nomuni kila ka me baleta na beka. Kevaka ko ni kila tale e so na I tukutuku me baleta na manumanu oqo, mo ni qai vakalewena e na vanua ko ni kila ni rawa ni vakacurumi kina. E na nomuni veivakadonui, na veitaro oqo ena katoni koto ena dua na monalivaliva lailai (voice recorder). Na veitalanoa e katoni, ena sega ni vakayagataki e na dua tani tale na ka. E na vakayagataki ga me ka ni vuli kei na vakadidike me baleti ira na beka, ka me vakadinadinataka ni veitarogi oqo e a yaco.*

8. Ni kemuni dau raica na beka, e na vanua o ni cakacaka se vaka I tikotiko kina?
   a. Io
   b. Sega
9. E vica kece na kena veimataqali ni manumanu oqo na beka o ni dau raica?
   _____________________________Comments: ___________________
10. Na cava o ni nanuma na kakana cava soti era dau kania na beka?
    a. Vua ni kau (fruits)
    b. Sore ni kau (seeds)
    c. Vu ni kau (plants)
    d. Manumanu lalai (insects)
    e. E so tale na ka? __________
11. Na cava o ni dau raica ni rad au kania na manumanu qo na beka?
    a. Vua ni kau (fruits)
    b. Sore ni kau (seeds)
    c. Vu ni kau (Plants)
    d. Manumanu lalai (insects)
    e. E so tale n aka ? _________
12. E na gauna cava soti oni dau raica vakalevu kina na beka? (gauna era veivukayaki, kana, vakavosavosa kina)
    a. Gauna ni cadra na siga (sunrise)
    b. Matakalailai (morning)
    c. Siga levu (afternoon)
    d. Yakavi/dromu na siga (sunset)
    e. Bogi (night)
    f. So tale na gauna? ________
13. E na nomu rai se kila ka, O ni nanuma na I wiliwili ni Beka e sa –
    a. Sa lailai tiko mai (decreasing)
    b. Sa levu tiko mai (increasing)
    c. Sega ni dua na veisau (toki sara ki na I kavitu (*15*) ni taro)
14. Na cava na nomu rai se nanuma e na vuku ni veisau ni wiliwili ni beka?
    a. Vakalevu tiko mai -
       _______________________
    b. Vakalailai tiko mai -
       _______________________
15. E na nomu rai, o ira na beka e:
    a. Vinaka? Baleta (vakamacala)
       _______________________
    b. Ca ? Baleta (Vakamacala)
       _______________________
    c. Vinaka ka ca talega (Kevaka ke a sega ni vakaleweni na taro e cake)
       _______________________
    d. Sega ni vinaka ka sega ni ca talega
       _______________________
16. Na cava o ni nanuma e nodra I tavi na beka e na veivanua?
    _____________________________
17. O ni nanuma ni o ira na beka era solia dua na vakacegu se vukea dua na tiki ni nomu bula e vei siga?
    a. Io – Vakamacalataka? _______________________
    b. Sega _______________________
18. O ni kila ni rawa beka vei ira na Beka me vakamavoataka na tamata?
    a. Io – Vakamacalataka? _______________________
    b. Sega, Vakamacalataka
       _______________________
19. Ocei ko ni nanuma era dau kania na Beka?
    __________________________________________________________

**Veikilai kei na veitaratara kei ira na Beka**

*Na veitaro ena muri mai ke e baleta na nomu veikilai, kila ka kei na veitaratara (Interactions) vata kei ira na manumanu qo na beka, ena tarogi tiko na nomuni dau toboka se na cava oni dau cakava kevaka oni sa toboka rawa e dua na beka, se na kena laukana. E so na taro e sa vakayagataki oti mai cake, kevaka e so na nomuni nanuma se sau ni taro e a sega ni tarogi ena veitaro sa oti, mo ni qai vakatakila toka ena veivanua lala e toka e ra. Kevaka oni sa vakalewena oti se sauma oti na veitaro I cake, ka koni sa sega ni rawa ni qai sauma tale na veitaro e ra oqo, ni qai tukuna cake mai.*

21. Ko ni kila ke tiko e so na beka ena vanua o ni vakatikotiko kina se na nomuni vanua ni cakacaka?
    a. Sega *- Lade sara kina I katolu (22) ni taro*
    b. Io
22. Na vanua cava soti era dau moce se vakacegu kina?
    a. Veivunikau
    b. Loma ni qaravatu
    c. E na ruku ni Wavu, vale lala?
    d. So tale na vanua? ________________
23. Sa bau turuvi kemuni oti na dra (Blood), weli (saliva), suasua (Urine) se valelailai (faeces) ni Beka?
    a. Sega
    b. Io _______________________
24. Sa bau vakamavoa taki kemuni oti na beka? (Kadrumi, laukati, etc)
    a. Sega
    b. Io _______________________
25. Ko ni a dau kana beka I liu?
    a. Sega - *Toki ki nai kaono (25) ni taro*
    b. Io
    c. E vica kece na kena levu oni sa kania oti? _______________________
    d. Dau va vica na nomu dau kania na beka?
       i. Vei siga
       ii. Vei Macawa
       iii. Vei Vula
       iv. Vei tolu na vula
       v. Vei ono na vula
       vi. Vei dua na yabaki
       vii. So tale na gauna? _________
    e. O ni a kauta/toboka mai vei na manumanu oqo?
       i. Toboka vakai-kemuni ga
       ii. Toboka vakalewe levu/lewe ni koro
       iii. Kauta/volia mai na makete?
       iv. So tale na vanua o ni a kauta mai kina ? _______________
    f. O ni a vakarautaka vakacava na kena caka/kana? _______________________
26. O ni se dau kania voliga na beka ena gauna oqo ?
    a. Sega - *Toki ki na I kavitu (26) ni taro*
    b. Io
    c. E vica kece na kena levu oni sa kania oti? _______________________
    d. Dau va vica na nomu dau kania na beka?
       i. Vei siga
       ii. Vei Macawa
       iii. Vei Vula
       iv. Vei tolu na vula
       v. Vei ono na vula
       vi. Vei dua na yabaki
       vii. So tale na gauna? __
    e. O ni a kauta/toboka mai vei na manumanu oqo?
       i. Toboka vakai-kemuni ga
       ii. Toboka vakalewe levu/lewe ni koro
       iii. Kauta/volia mai na makete?
       iv. So tale na vanua o ni a kauta mai kina ? _______________
    f. O ni a vakarautaka vakacava na kena caka/kana?
       _______________________
27. E na gauna I LIU, ko ni a dau vakayagataka na manumanu qo na Beka e na so tale na ka, duidui mai na kena e laukana?
    a. Sega - *lade kina I kawalu(27) ni taro*
    b. Io. Rawa ni ko vakamacalataka nomu sau ni taro. _______________________
    c. E vica kece na kena levu oni sa vakayagataka oti? _______________________
    d. Dau va vica na nomu dau kania na beka?
       i. Vei siga
       ii. Vei Macawa
       iii. Vei Vula
       iv. Vei tolu na vula
       v. Vei ono na vula
       vi. Vei dua na yabaki
       vii. So tale na gauna? _________
    e. O ni a kauta/toboka mai vei na manumanu oqo?
       i. Toboka vakai-kemuni ga
       ii. Toboka vakalewe levu/lewe ni koro
       iii. Kauta/volia mai na makete?
       iv. So tale na vanua o ni a kauta mai kina ? ______________
    f. O ni a vakarautaka vakacava na kena caka/kana?
       _______________________
28. E na gauna OQO, Ko ni se vakayagataka voliga na beka me kani so tale na ka, duidui mai na kena e laukana?
    a. Sega - *Lade ki na I kaciwa(28) ni taro*
    b. Io - vakamacalataka ______
    c. E vica kece na kena levu oni sa toboka oti? _______________________
    d. Dau va vica na nomu dau toboka na beka?
       i. Vei siga
       ii. Vei Macawa
       iii. Vei Vula
       iv. Vei tolu na vula
       v. Vei ono na vula
       vi. Vei dua na yabaki
       vii. So tale na gauna? _________
    e. O ni a kauta/toboka mai vei na manumanu oqo?
       i. Toboka vakai-kemuni ga
       ii. Toboka vakalewe levu/lewe ni koro
       iii. Kauta/volia mai na makete?
       iv. So tale na vanua o ni a kauta mai kina ? _______________
    f. O ni a vakarautaka vakacava na kena caka/kana?
       _______________________
29. O ni a dau tobo Beka vakai iko ena gauna I liu?
    a. Sega - *Lade ki nai katini (29) ni taro*
    b. Io - yabaki ko ni a sa toboka oti kina e dua? ______________________
    c. Ko nia dau toboki ira ena veigauna se e na gauna ga o raici ira kina?
       _______________________
    d. E vica kece ko ni a dau toboka ena dua na gauna?
       _______________________
    e. O ni dau toboka ena vei:
       i. Siga
       ii. Macawa
       iii. Vula
       iv. Tolu na macawa
       v. Ono na macawa
       vi. Dua na yabaki
       vii. So tale na gauna? _________________
    f. Ke na I naki?
       i. Me laukana?
       ii. Me I vurevure ni Lavo
       iii. Me kani soli se loloma
       iv. So tale na kena yakayagataki? _________________
30. O ni se tobo beka tikoga e na gauna oqo?
    a. Sega - *Lade ki na I katinikadua(30) ni taro*
    b. Io
    c. Ko nia dau toboki ira ena veigauna se e na gauna ga o raici ira kina?
       _______________________
    d. E vica kece ko ni a dau toboka ena dua na gauna?
       _______________________
    e. O ni dau toboka ena vei:
       i. Siga
       ii. Macawa
       iii. Vula
       iv. Tolu na macawa
       v. Ono na macawa
       vi. Dua na yabaki
       vii. So tale na gauna? _________________
    f. Ke na I naki?
       i. Me laukana?
       ii. Me I vurevure ni Lavo
       iii. Me kani soli se loloma
       iv. So tale na kena yakayagataki? _________________
31. Ni koni dau vakarautaka na Beka me ka ni laukana?
    a. Sega - *Lade ki na I katinikarua(31) ni taro*
    b. Io
    c. E vica kece ko ni dau vakarautaka? _______________________
    d. O ni dau vakarautaka ena vei:
       i. Siga
       ii. Macawa
       iii. Vula
       iv. Tolu na macawa
       v. Ono na macawa
       vi. Dua na yabaki
       vii. So tale na gauna? _________________
    e. Oni dau cakava vakacava na kena vakarautaki? _______________________
32. O ni sa bau vakayagataka na nodra valelailai se de ni beka ena gauna I liu?
    a. Sega - *Lade ki na I katinikatolu (32) ni taro*
    b. Io
    c. Dau vakacava na kena I vakarau oni dau vakayagataka? ______________
    d. O ni dau vakayagataka ena vei:
       i. Siga
       ii. Macawa
       iii. Vula
       iv. Tolu na macawa
       v. Ono na macawa
       vi. Dua na yabaki
       vii. So tale na gauna? _________________
    e. Vanua e kau mai kina?
       i. O ni kauta se vakarautaka vakai kemuni ga.
       ii. O ni volia mai
       iii. So tale na vanua o ni kauta main a nodra vale-lailai se de ni beka.? _________________
    f. Na kena vakayagataki?
       i. Nomu I vakayagataka ena vukumu ga (personal use)
       ii. Me I vurevure ni I lavo
       iii. Me ka ni soli se I loloma.
       iv. So tale na kena I vakayagataki koni kila _________________
33. O ni se dau vakayagataka voliga na nodra vale-lailai se na de ni beka ena gauna oqo?
    a. Io
    b. Sega - *Lade ki na I katinikava (33) ni taro*
    c. Dau vakacava na kena I vakarau oni dau vakayagataka? ______________
    d. O ni dau vakayagataka ena vei:
       i. Siga
       ii. Macawa
       iii. Vula
       iv. Tolu na macawa
       v. Ono na macawa
       vi. Dua na yabaki
       vii. So tale na gauna? _________
    e. Vanua e kau mai kina?
       i. O ni kauta se vakarautaka vakai kemuni ga.
       ii. O ni volia mai
       iii. So tale na vanua o ni kauta main a nodra vale-lailai se de ni beka.? _________________
    f. Na kena vakayagataki?
       i. Nomu I vakayagataka ena vukumu ga (personal use)
       ii. Me I vurevure ni I lavo
       iii. Me ka ni soli se I loloma.
       iv. So tale na kena I vakayagataki koni kila _________________
34. O ni dau lakova se sikova na nodra I vunivuni na beka?
    a. Io
    b. Sega - *Lade ki na I katinikalima(34) ni taro.*
    c. Era dau vuni se vakacegu vakalevu I na vanua cava o ira na beka oqo. (qaravatu, vu-ni-kau, ruku ni wavu, etc) _______________________
    d. Na cava na vuni nomuni dau sikova kina na nodra vanua ni vakavakacegu? __________________
       i. O ni dau sikova ena vei:
          Siga
       ii. Macawa
       iii. Vula
       iv. Tolu na macawa
       v. Ono na macawa
       vi. Dua na yabaki
       vii. So tale na gauna? _________________
35. Kevaka o ni raica e dua na beka e mavoa tu se tauvimate – na cava o ni na cakava?
    a. Laiva vakadua
    b. Vakayavalata
    c. Qarava me vinaka na manumanu
    d. Vakamatea
    e. Volitaka
    f. Vakayagataka me laukana
    g. So tale na ka. ___________________
36. E so tale na ka o ni dau cakava kei ira na manumanu qo, na Beka?

Io se sega

Kerekere vakamacalataka mada.

__________________________________________________________

37. Kevaka e so tale na I tukutuku me baleti ira na manumanu qo na beka, e a sega ni tarogi, se a vakalutumi ena gauna ni veitarogi qo, qai kerea moni vakamacalataka mada mai.
    ___________________________________________________________________________________________________________________________________________________________________________________________________________

Vinaka vakalevu, na nomuni taura tiko na nomuni gauna mo ni vakalewena e dua na ka ni vakadidike lailai oqo, au sa nuitaka na nomuni veitokoni kei na nomuni yalodina ena veisaumi taro.

*Table S1: Participant details - Gender, and age category*

| **Participant details** | **Gender** | | **Age category (in years)** | | | | | |
| --- | --- | --- | --- | --- | --- | --- | --- | --- |
|  | Male | Female | 18-24 | 25-29 | 30-39 | 40-49 | 50-59 | 60+ |
| **Total** | 108 | 90 | 31 | 33 | 47 | 32 | 28 | 27 |
| **Percentage** | 54.5 | 45.5 | 15.7 | 16.7 | 23.7 | 16.2 | 14.1 | 13.6 |

*Table S2: Participant details – education in years, where NA = No Answer*

| **Participant details** | **Education (in years)** | | | | | | | | | | | | |  |
| --- | --- | --- | --- | --- | --- | --- | --- | --- | --- | --- | --- | --- | --- | --- |
|  | 3 | 4 | 5 | 6 | 7 | 8 | 9 | 10 | 11 | 12 | 13 | 13+ | NA | |
| **Total** | 2 | 1 | 1 | 4 | 3 | 26 | 14 | 31 | 22 | 48 | 10 | 32 | 4 | |
| **Percentage** | 1 | 0.5 | 0.5 | 2 | 1.5 | 13.1 | 7.1 | 15.7 | 11.1 | 24.2 | 5.1 | 16.2 | 2 | |

*Table S3: Participant details - religion, where NA = No Answer*

| **Participant details** | **Religion** | | | | | | |
| --- | --- | --- | --- | --- | --- | --- | --- |
|  | Methodist | Christian | Catholic | Other Christian | Hindu | No religion | NA |
| **Total** | 102 | 23 | 35 | 27 | 6 | 2 | 3 |
| **Percentage** | 51.5 | 11.6 | 17.7 | 13.6 | 3 | 1 | 1.5 |

*Table S4: Reported presence of bat roosts close to human activity*

| **Question** | **Are there any bats roosting near where you live, commute or work?** | | **If YES, Where are the roosts?** | | | |
| --- | --- | --- | --- | --- | --- | --- |
|  | Yes | No | Tree | Cave | Tree and cave | NA |
| **Response** | 128 | 69 | 114 | 3 | 11 | 69 |
| **Percentage** | 65 | 35 | 89.1 | 2.3 | 8.6 | - |

Note: NA = Not applicable as no roosting reported

*Table S5: Participant perceptions about the diet of bats and what they have been observed to eat*

| **Question** | **What do you think bats eat?** | | | | | | **What have you seen bats eat?** | | | | | |
| --- | --- | --- | --- | --- | --- | --- | --- | --- | --- | --- | --- | --- |
|  | Fruit | Seed | Plants/ Flowers | Insects | Other^#^ | NA | Fruit | Seed | Plants/ Flowers | Insects | Other^$^ | NA |
| **Number** | 194 | 6 | 27 | 14 | 8 | 2 | 195 | 3 | 14 | 9 | 5 | 1 |
| **Comments** |  | 5/6* | 26/27* | 12/14* | 5/8*, 2/8** | No Answer |  | 2/3* | 13/14* | 7/9* | 1/5*, 1/5** | No Answer |
| ^#^Other = lizards, rats, nuts, dew/mist, ground fruit  ^$^Other = dew/mist, nuts  * also answered Fruit  ** also answered Insects | | | | | | | | | | | | |

*Table S6: Reported time of day for bat activity*

| **Question** | **At what times do you see bats being active?** | | | | | |
| --- | --- | --- | --- | --- | --- | --- |
|  | Dawn/ Sunrise | Morning | Afternoon | Dusk/ Sunset | Night | Other* |
| **Number** | 20 | 9 | 85* | 45* | 113 | 13 |
| **Comments** | 86 participants answered 2 or more times of day that bats were active. Some answers varied depending on size/type of bat.  *Other: “during day go up and down in the trees above the mountain”; “if clouded weather will be out active at lunchtime”; “tan ones are seen at any time of day”; “if disturbed will see during the day”; “B. lulu anytime during the day”; “only see during a cyclone”. | | | | | |

*Table S7: Logistic regression model of contact between people and bats, including recognition that bats can harm humans, without age and gender interactions*

| **Coefficients** | **Estimate (S.E.)** | **p-value** |
| --- | --- | --- |
| (Intercept) | −2.70 (0.56) | <0.001 |
| Recognises bats can harm humans | 1.44 (0.36) | <0.001 |
| Age 25–29 | 0.34 (0.60) | 0.57 |
| Age 30–39 | 0.94 (0.56) | 0.09 |
| Age 40–49 | 1.52 (0.62) | 0.01 |
| Age 50–59 | 1.34 (0.62) | 0.03 |
| Age 60+ | 2.61 (0.72) | <0.001 |
| Male | 1.69 (0.36) | <0.001 |

*Estimates are log-odds from binomial logistic regression models. Age reference category is 18–24 years; gender reference category is female. “Recognises bats can harm humans” is coded as a binary variable derived from participant responses.*

*Table S8: Logistic regression model of current bat meat consumption, including recognition that bats can harm humans, without age and gender interactions*

| **Coefficients** | **Estimate (S.E.)** | **p-value** |
| --- | --- | --- |
| (Intercept) | −1.35 (0.47) | 0.004 |
| Recognises bats can harm humans | 0.11 (0.31) | 0.72 |
| Age 25–29 | 1.08 (0.54) | 0.047 |
| Age 30–39 | 0.56 (0.50) | 0.26 |
| Age 40–49 | 1.59 (0.55) | 0.004 |
| Age 50–59 | 1.22 (0.57) | 0.03 |
| Age 60+ | 1.51 (0.58) | 0.009 |
| Male | 0.74 (0.31) | 0.02 |

*Estimates are log-odds from binomial logistic regression models. Age reference category is 18–24 years; gender reference category is female. “Recognises bats can harm humans” is coded as a binary variable derived from participant responses.*

*Table S9: Logistic regression model of past bat meat consumption, including recognition that bats can harm humans, without age and gender interactions*

| **Coefficients** | **Estimate (S.E.)** | **p-value** |
| --- | --- | --- |
| (Intercept) | 0.18 (0.49) | 0.72 |
| Recognises bats can harm humans | 0.73 (0.38) | 0.06 |
| Age 25–29 | −0.02 (0.60) | 0.97 |
| Age 30–39 | 0.30 (0.56) | 0.59 |
| Age 40–49 | 0.51 (0.64) | 0.43 |
| Age 50–59 | 0.13 (0.65) | 0.84 |
| Age 60+ | 2.17 (1.12) | 0.05 |
| Male | 1.04 (0.40) | 0.008 |

*Estimates are log-odds from binomial logistic regression models. Age reference category is 18–24 years; gender reference category is female. “Recognises bats can harm humans” is coded as a binary variable derived from participant responses.*

*Table S10: Logistic regression model of the preparation of bats as food, including recognition that bats can harm humans, without age and gender interactions*

| **Coefficients** | **Estimate (S.E.)** | **p-value** |
| --- | --- | --- |
| (Intercept) | −0.62 (0.44) | 0.16 |
| Recognises bats can harm humans | 0.42 (0.33) | 0.21 |
| Age 25–29 | −0.13 (0.52) | 0.81 |
| Age 30–39 | 1.14 (0.49) | 0.02 |
| Age 40–49 | 1.51 (0.57) | 0.007 |
| Age 50–59 | 0.74 (0.56) | 0.18 |
| Age 60+ | 2.72 (0.82) | <0.001 |
| Male | 0.33 (0.34) | 0.33 |

*Estimates are log-odds from binomial logistic regression models. Age reference category is 18–24 years; gender reference category is female. “Recognises bats can harm humans” is coded as a binary variable derived from participant responses.*
